# Polymyxin-resistant hypervirulent *Klebsiella pneumoniae* clinical isolates from a tertiary hospital in China: molecular mechanisms, antimicrobial susceptibility, and virulent phenotypes

**DOI:** 10.3389/fmicb.2025.1705471

**Published:** 2025-12-17

**Authors:** Xinyu Zhu, Jie Yan, Xiyun Lai, Tingjian Zou, Mengyuan Chen, Xiaofang Tang, Qiang Wang, Yuzhou He

**Affiliations:** 1 The Second Clinical Medical College of Zhejiang Chinese Medical University, Hangzhou, China; 2 Department of Clinical Laboratory, The Second Affiliated Hospital of Zhejiang Chinese Medical University, Hangzhou, China; 3 Department of General Medicine, Zhejiang Hospital, Hangzhou, China; 4 Department of Emergency, The Second Affiliated Hospital of Zhejiang Chinese Medical University, Hangzhou, China

**Keywords:** hypervirulent, *mgrB*, *PhoPQ*, polymyxin-resistant *Klebsiella pneumoniae*, virulence

## Abstract

**Background:**

Polymyxins are critical last-resort treatments for infections caused by multidrug-resistant (MDR) gram-negative bacteria, especially carbapenem-resistant isolates. Recently, there has been a continuous increase in reports of polymyxin-resistant *Klebsiella pneumoniae* (PoRKP), which poses a major healthcare challenge. This study aims to investigate the current epidemiological trends and virulent phenotypes of PoRKP and to elucidate the mechanisms underlying polymyxin resistance.

**Methods:**

In this study, 17 PoRKP strains were retrospectively identified from 2,146 *Klebsiella pneumoniae* (KP) clinical isolates collected in Hangzhou city from 2018 to 2021. We assessed the antimicrobial resistance and virulence profiles of 17 isolates, and they were subjected to molecular and genetic analyses and antimicrobial susceptibility testing.

**Results:**

The overall polymyxin resistance rate among the bacterial isolates included in this study was low (0.8%); however, all PoRKP isolates were MDR-positive. Notably, only three isolates carried *mcr*, whereas the vast majority either harbored mutations in *mgrB* (C28R or C28Y) or *pmrA* (T246A and R256G) leading to overexpression of the two-component systems, which we consider to be the primary mechanism underlying polymyxin resistance. Furthermore, five PoRKP isolates carrying hypervirulence genes exhibited a virulent phenotype in a *Galleria mellonella* infection model. Finally, recent antibiotic therapy, invasive procedures, and age were identified as important risk factors for the occurrence of polymyxin-resistant strains.

**Conclusion:**

Our findings indicated a relatively low prevalence of plasmid-mediated mobile polymyxin resistance genes among PoRKP isolates, whereas the upregulation of the two-component systems (particularly phoPQ) played a more prominent role in mediating polymyxin resistance.

## Introduction

Antimicrobial resistance is a major global concern. The lack of early identification of the causative organisms and their antimicrobial (Aggarwal et al., 2024) susceptibility patterns has led to widespread and often unnecessary use of broad-spectrum antibiotics since the 1990s and has driven the emergence of numerous drug-resistant bacteria. Additionally, the unregulated management of nosocomial infections has increased the risk of transmission of drug-resistant bacteria, especially drug-resistant Enterobacteriaceae (Romanescu et al., 2023). In 2017, the World Health Organization added multidrug-resistant Enterobacteriaceae to its list of key pathogens (Mancuso et al., 2021).

*Klebsiella pneumoniae* (KP) is a gram-negative bacterium belonging to the family Enterobacteriaceae and often causes respiratory tract infections and is commonly associated with hospital-acquired pneumonia (Prince et al., 1997; Wong et al., 2022; Zhong et al., 2021). Of particular concern is the emergence of hypervirulent *Klebsiella pneumoniae* (hvKP), which can cause severe community-acquired infections in healthy people and has been associated with metastatic infections (Al et al., 2024) such as liver abscess, meningitis, and endophthalmitis (Pu et al., 2023; Singh et al., 2024). The prevalence of hypervirulent *Klebsiella pneumoniae* and multidrug-resistant *Klebsiella pneumoniae* (MDR-KP) has increased in recent years, including the emergence of polymyxin-resistant *Klebsiella pneumoniae* (PoRKP) strains (Russo and Marr, 2019). This trend has greatly narrowed effective therapeutic options, considerably increased treatment complexity, and posed severe challenges to clinical antimicrobial therapy and public health prevention and control (Szymanski et al., 2024).

Previous studies have rarely conducted a combined analysis of drug resistance and virulence, and hypervirulent PoRKP is relatively uncommon. In this study, we retrospectively identified 17 PoRKP strains from 2,146 non-repetitive clinical KP isolates collected in Hangzhou from 2018 to 2021. Molecular and genetic analyses revealed that chromosome-mediated resistance is the predominant mechanism underlying PoRKP. Accordingly, we investigated the effect of transcription and genetic variations of genes involved in two-component systems (TCSs) and their regulators on polymyxin resistance. Virulence phenotype was determined in 17 PoRKPs. These findings provide evidence that the upregulated expression of TCSs (especially phoPQ) contributes substantially to polymyxin resistance development. The potential for high-level polymyxin-resistant MDR-KP to become hypervirulent highlights the importance of monitoring both resistance mechanisms and virulence factors in PoRKP.

## Methods

### Strains and antimicrobial susceptibility testing

A total of 2,146 non-duplicate KP isolates were collected from patients hospitalized at the Zhejiang Chinese Medical University Hospital in Hangzhou, eastern China, between 2018 and 2021. All isolates were identified using matrix-assisted laser desorption/ionisation time-of-flight mass spectrometry (Autof ms1500, Autobio, Zhengzhou, China) and were further confirmed by 16S rRNA sequencing (Johnson et al., 2019; Paul, 2022). The minimum inhibitory concentrations (MICs) of 16 antibiotics against the PoRKP isolates, except for tigecycline, were determined using the broth microdilution method as defined by the Clinical and Laboratory Standards Institute (CLSI, 2025). MIC breakpoints of tigecycline for the PoRKP isolates (susceptible, ≤2 mg/L; resistant, ≥8 mg/L) were issued by the Food and Drug Administration. This study has been approved by the Ethics Committee of the Second Affiliated Hospital of Zhejiang Chinese Medical University (ethical approval no.: Y2024-037-01).

### Multi-locus sequence typing (MLST) and K antiserum typing (serotypes)

Primers for seven housekeeping genes (*rpoB, gapA, mdh, pgi, phoE, infB, tonB*) were designed according to the guidelines provided on the MLST website^1^. PCR amplification was carried out under the following conditions: initial denaturation at 94 °C for 3 min, followed by 34 cycles of denaturation at 98 °C for 10 s, annealing at 55 °C for 30 s, and extension at 72 °C for 2 min, with a final extension at 72 °C for 5 min. Amplified products were visualized on a 1% agarose gel (Wittmeier and Hummel, 2022). Sanger sequencing of the amplified products was performed by Youkang Biotechnology Co., Ltd. (Hangzhou, China). Based on the obtained sequences, sequence types (STs) were determined through the MLST website^2^.

K antiserum typing (serotyping) was performed according to the aforementioned PCR procedures (Supplementary Table S1). Sanger sequencing of the amplified products was performed by Youkang Biotechnology Co., Ltd. (Hangzhou, China). Based on the obtained sequences, Nucleotide sequences were compared using BLAST^3^.

### Identification of genes corresponding resistance to polymyxin

Using gene-specific primer pairs, we assessed the presence or absence of the following genes in the clinical isolates of *K. pneumoniae*: *mgrB, phoP, phoQ, pmrA, pmrB* and *mcr* (Supplementary Table S2). Primer specificity was verified using the primer BLAST program on the NCBI server. Each gene was amplified separately in 50 μL PCR reaction mixtures, and the PCR products were identified based on fragment size and visualized on a 1% agarose gel. Sanger sequencing of the amplified products was performed by Youkang Biotechnology Co., Ltd. (Hangzhou, China).

### Relative expression of drug resistance genes

To explore the relationship between the regulons and drug resistance (Bong et al., 2024; Rocha et al., 2020), we analyzed the expression of polymyxin resistance genes. Seventeen polymyxin-resistant strains were classified into the polymyxin-resistant group, while 17 strains of clinically isolated polymyxin-sensitive KP were used as controls. Briefly, all isolates were grown to the logarithmic phase, and total RNA was extracted using the SteadyPure Quick RNA Extraction Kit (Accurate Biotechnology, Hunan, China). The total RNA of each isolate was reverse transcribed into cDNA using the Evo M*-*MLV RT Mix Kit (with gDNase) (Accurate Biotechnology). Finally, gene expression data were obtained by reverse transcription quantitative real-time PCR (RT-qPCR) using the CFX96™ Real-Time System (Bio-Rad, USA) and the SYBR Green Premix Pro Taq HS qPCR Kit (Rox Plus) (Accurate Biotechnology); *gapdh* served as an internal reference gene. Each experiment was repeated three times, and the relative expression of each gene in different strains was calculated using the 2^-ΔΔCt^ method (Bong et al., 2024).

### Whole-genome sequencing

Bacterial DNA was extracted, its concentration quantified using a NanoDrop™ 2000 spectrophotometer (Thermo Scientific, Waltham, MA, USA), and purity verified by agarose gel electrophoresis. A minimum of 50 ng of DNA was required for library preparation prior to next-generation sequencing (Dalwadi et al., 2024; Parvez et al., 2020).

Libraries were prepared using the TruePrep™ DNA Library Prep Kit V2 for Illumina (Vazyme), and subsequently sequenced on an Illumina NovaSeq platform (Illumina Inc., San Diego, CA, USA), which generated 150 bp paired-end reads. The sequenced raw reads underwent quality checks using Fastqc (version 0.11.9) and Multiqc (version 1.10.1). Trimmed reads were assembled using the *de novo* assembly (primary assembly) tool Unicycler (v0.4.5)^4^. Functional annotation was performed using the NCBI protein database, non-redundant protein sequences (NR), SwissProt^5^, Kyoto Encyclopedia of Genes and Genomes (KEGG)^6^, and Clusters of Orthologous Groups of proteins (COG)^7^.

The Resistance Gene Identifier (RGI) 4.0.3 software was utilized to annotate resistance genes based on the Comprehensive Antibiotic Resistance Database (CARD).

### SNP calling and phylogeny reconstruction

The publicly available genome of KP NTUH-K2044 (Zulkifli et al., 2013) (accession no.: NC_012731.1) was used as a reference (Zarras et al., 2023). Additionally, 11 PoRKP isolates with the same mutation profiles were downloaded from the NCBI database for comparative analysis. All genomes were aligned against the reference genome using MAFFT (v7.471) to generate whole-genome alignments and identify single nucleotide polymorphisms (SNPs) in the core genome, with repetitive regions removed. Based on the concatenated SNPs, a maximum likelihood tree was constructed using IQ-TREE (v1.6.12) with default parameters.

### Identification of virulence genes

PCR amplification, electrophoresis, and product sequencing were performed according to the reaction conditions described above. Using gene-specific primer pairs, we identified the presence of virulence genes such as *rmpA*, *rmpA_2_*, *iucA*, *peg344*, *iroB*, and *aerobactin* in the clinical isolates of PoRKP (Supplementary Table S3).

### String test

String tests were conducted as previously described (Li et al., 2020; Yao et al., 2015). All isolates were cultured on Columbia Blood Agar plates (BIO-KONT, Wenzhou, China) incubated overnight at 37 °C. Using an inoculation loop, the colonies were gently touched and lifted. A positive test result was defined by the formation of a viscous string typically exceeding 5 mm in length.

### Virulence testing using the *Galleria mellonella* infection model

We estimated PoRKP’s virulence and pathogenicity by infecting *Galleria mellonella* larvae (Chen et al., 2021; McLaughlin et al., 2014). NTUH-K2044 was used as the hypervirulent control strain, and *Klebsiella pneumoniae* K6 (ATCC 700603), which lacks virulence genes, served as the low-virulence control strain. Overnight cultures of KP were diluted in sterile phosphate-buffered saline to obtain a concentration of 10^8^ CFU/mL. *G. mellonella* larvae weighing 250–300 mg (Henan Jiyuan Baiyun Industry Co., Ltd., Henan, China) were injected with 10 μL bacterial suspension and incubated for 72 h at 35 °C. The survival rate of *G. mellonella* was recorded at 12, 24, 36, 48, 60, and 72 h. Ten larvae were tested per isolate, and all experiments were performed in triplicate. Kaplan–Meier survival curves were plotted using the GraphPad Prism software (10.1.2). The survival rate results are presented as the range of data from three independent experiments.

### Statistical analysis

The linear correlation between the transcript levels of chromosomal regulators and polymyxin MICs was analyzed using GraphPad Prism 8. The Mann–Whitney U test and Spearman’s rank correlation were performed using SPSS v25.0. Mantel–Haenszel χ^2^ test was used to assess the association between virulence and MICs. The log-rank test (Mantel-Cox) was used to analyze the survival curve of the *G. mellonella* infection models using GraphPad Prism 8. A *p*-value < 0.05 was regarded to be statistically significant, while a *p-*value < 0.001 was considered extremely significant.

## Results

### Clinical information and analysis

**Figure 1:**
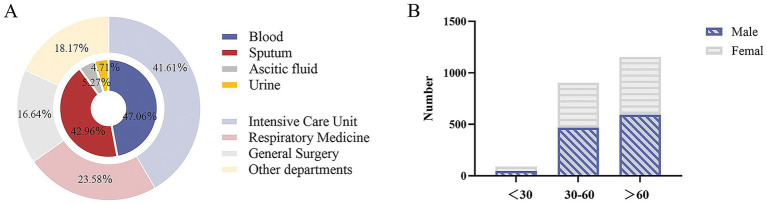
Clinical information on 2,146 *Klebsiella pneumoniae.* **(A)** Strain source department and specimen type. **(B)** Age and gender distribution of patients infected with *Klebsiella pneumoniae*.

Between 2018 and 2021, a total of 2,146 non-repetitive clinical KP isolates were collected from blood, 47.06% (1,010/2146); sputum, 42.96% (922/2146); ascitic fluid, 5.27% (113/2146); and urine, 4.71% (101/2146). The isolates were obtained from various hospital departments, including the intensive care unit (41.61%, 893/2146), respiratory medicine (23.58%, 506/2146), general surgery (16.64%, 357/2146), and other departments (e.g., hematology, nephrology, and geriatrics) (18.17%, 390/2146). The age of the patients ranged from 3 to 102 years; patients >60 years accounted for 53.73% (1,153/2146), those aged 30–60 years made up 42.08% (903/2146), and <30 years accounted for 4.19% (90/2146) (Figure 1).

Recent antibiotic therapy (within 1 month before the isolation of PoRKP), invasive procedures, and age were identified as important risk factors for the emergence of polymyxin-resistant strains in clinical settings (Table 1).

**Table 1:** Risk factors associated with clinical characteristics caused by Polymyxin-resistant *Klebsiella pneumonia* (PoRKP) isolates.

Risk factors	*K. pneumoniae* Isolates (*n* = 2,146)				*χ*2	*P*
	PoR *K. pneumoniae*		Non PoR *K. pneumoniae*			
	(*n* = 17)		(*n* = 2,129)			
	*n*	%	*n*	%		
Age						
<70 (*n* = 1,297)	4	23.5%	1,293	60.7%	9.76	0.002*
≥70 (*n* = 849)	13	76.5%	836	39.3%		
Previous hospitalization of 5 days or more						
Yes (*n* = 1,033)	12	70.6%	1,021	48.0%	3.46	0.062
No (*n* = 1,113)	5	29.4%	1,108	52.0%		
Duration of mechanical ventilation						
<48 h (*n* = 1,102)	10	58.8%	1,092	51.3%	0.38	0.536
≥48 h (*n* = 1,044)	7	41.2%	1,037	48.7%		
Invasive procedures						
Yes (*n* = 1,233)	16	94.1%	1,217	57.2%	9.42	0.002*
No (*n* = 913)	1	5.9%	912	42.8%		
Recent antibiotics intake						
Yes (*n* = 941)	15	88.2%	926	43.5%	13.71	0.000*
No (*n* = 1,205)	2	11.8%	1,203	56.5%		

*Statistically significant at *p* < 0.05.

### Antibiotic susceptibility test results

Antimicrobial susceptibility testing was performed on the 17 PoRKP strains. The prevalence of PoRKP was 0.79% (*n* = 17/2146). The polymyxin MICs for the strains ranged from 4 to 64 mg/L. All PoRKP strains exhibited similar antimicrobial susceptibility profiles, showing resistance to most antibiotics tested (Piperacillin/Tazobactam, Ciprofloxacin, Levofloxacin, Cefuroxime, Ceftriaxone, Cefepime, Aztreonam, Gentamicin, Amikacin, Cefoxitin, Imipenem, Meropenem) but were susceptible to ceftazidime/avibactam. Accordingly, all 17 strains were MDR, with 14 identified as extensively drug-resistant (XDR) (Table 2).

**Table 2:** Antimicrobial resistance of clinical PoRKP isolates (MICs, mg/L).

Strains	Specimen types	Antimicrobial agent																
		PB	CAZ	CZA	TGC	TZP	CIP	LEV	CXM	CRO	FEP	ATM	GEN	AMK	AMC	FOX	IPM	MEM
PB1	Blood	64	≥64	≤2	0.5	≥128	≥4	≥8	≥64	≥64	≥64	≥64	≥16	≥64	≥32	≥64	≥16	≥16
PB2	Blood	64	≥64	2	1	≥128	≥4	≥8	≥64	≥64	≥64	≥64	≥16	≥64	≥32	≥64	≥16	≥16
PB3	Blood	4	≥64	64	4	≥128	≥4	≥8	≥64	≥64	≥32	≥64	≥16	≥64	≥32	≥64	≥16	≥16
PB4	Blood	16	≥64	2	0.5	≥128	≥4	≥8	≥64	≥64	≥64	≥64	≥16	≥64	≥32	≥64	≥16	≥16
PB5	Blood	64	≥64	2	4	≥128	1	1	≥64	16	2	≥64	≥16	≤2	≥32	16	≥16	≥16
PB6	Blood	32	≥64	2	4	≥128	≥4	≥8	≥64	≥64	≥32	≥64	≥16	8	≥32	≥64	≥16	≥16
PB7	Sputum	16	≥64	2	2	≥128	≥4	≥8	≥64	≥64	≥64	≥64	≥16	≥64	≥32	≥64	≥16	≥16
PB8	Sputum	8	≥64	2	≥8	≥128	≥4	≥8	≥64	≥64	≥64	≥64	≥16	≥64	≥32	≥64	≥16	≥16
PB9	Blood	16	≥64	2	4	≥128	≥4	≥8	≥64	≥64	≥64	≥64	≥16	≤2	≥32	≥64	≥16	≥16
PB10	Urine	64	≥64	2	2	≥128	≥4	≥8	≥64	≥64	≥64	≥64	≥16	≥64	≥32	≥64	≥16	≥16
PB11	Sputum	64	≥64	2	1	≥128	≥4	≥8	≥64	≥64	≥64	≥64	≥16	≥64	≥32	≥64	≥16	≥16
PB12	Blood	8	≥64	2	0.5	≥128	≥4	≥8	≥64	≥64	≥64	≥64	≥16	≥64	≥32	≥64	≥16	≥16
PB13	Blood	4	≥64	2	4	≥128	≥4	≥8	≥64	≥64	≥64	≥64	8	≥64	≥32	≥64	4	≥16
PB14	Ascitic fluid	4	≥64	2	1	≥128	≥4	≥8	≥64	≥64	≥64	≥64	≥64	≥64	≥32	≥64	8	≥16
PB15	Sputum	32	≥64	≤2	4	≥128	≥4	≥8	≥64	≥64	≥32	≥64	≥16	≥64	≥32	≥64	2	≥16
PB16	Sputum	32	≥64	2	4	≥128	≥4	≥8	≥64	≥64	≥32	≥64	20	≤2	≥32	≥64	≥16	≥16
PB17	Sputum	32	≥64	2	4	≥128	≥4	≥8	≥64	≥64	≥32	≥64	12	32	≥32	≥64	4	≥16

PB, Polymyxin B; CAZ, Ceftazidime; CZA, Ceftazidime/Avibactam; TGC, Tigecycline; TZP, Piperacillin/Tazobactam; CIP, Ciprofloxacin; LEV, Levofloxacin; CXM, Cefuroxime; CRO, Ceftriaxone; FEP, Cefepime; ATM, Aztreonam; GEN, Gentamicin; AMK, Amikacin; AMC, Amoxicillin/clavulanic acid; FOX, Cefoxitin; IPM, Imipenem; MEM, Meropenem.

### MLST and K type

Genotyping was performed on the 17 PoRKP strains (Table 3); all belonged to either sequence type 11 (ST11) (47.1%, 8/17) (PB1, PB2, PB3, PB5, PB10, PB11, PB12, PB16) or sequence type 15 (ST15) (52.9%, 9/17) (PB4, PB6, PB7, PB8, PB9, PB13, PB14, PB15, PB17). The capsular serotypes were K19 (17.6%, 3/17) (PB3, PB6, PB9), K21 (23.5%, 4/17) (PB10, PB11, PB15, PB17), K64 (29.4%, 5/17) (PB1, PB2, PB5, PB12, PB16), and KL112 (29.4%, 5/17) (PB4, PB7, PB8, PB13, PB14).

**Table 3:** Key results summary table.

Strains	MLST	K Types	Resistance-related genetic mutations	Virulence potential		
				Virulence genes	String test	Survival rate of *Galleria* model
PB1	ST11	K64	*PmrB* (T246A and R256G)	*iucA*, *rmpA*, *rmpA_2_*, *peg344*	+	< 20%
PB2	ST11	K64	/	*iucA*, *rmpA*, *rmpA_2_*, *peg344*	−	60–70%
PB3	ST11	K19	*PmrB* (T246A and R256G)	/	−	70–80%
PB4	ST15	KL112	/	*iucA*, *rmpA_2_*, *peg344*	−	30–40%
PB5	ST11	K64	*PmrB* (T246A and R256G)	*iucA*, *rmpA*, *rmpA_2_*, *peg344*	−	20–30%
PB6	ST15	K19	/	/	−	60–70%
PB7	ST15	KL112	*mgrB* (C28R)	*iucA*, *rmpA_2_*	−	<20%
PB8	ST15	KL112	*mgrB* (C28R)	*iucA*, *rmpA_2_*, *peg344*	−	<20%
PB9	ST15	K19	/	/	−	70–80%
PB10	ST11	K21	*PmrB* (T246A and R256G)	*iucA*, *rmpA*, *rmpA_2_*, *peg344*	−	60–70%
PB11	ST11	K21	*PmrB* (T246A and R256G)	*iucA*, *rmpA*, *rmpA_2_*	−	60–70%
PB12	ST11	K64	*PmrB* (T246A and R256G)	*iucA*, *rmpA*, *rmpA_2_*, *peg344*	+	<20%
PB13	ST15	KL112	/	*iucA*, *rmpA_2_*	+	<20%
PB14	ST15	KL112	*mgrB* (C28Y)	*iucA*, *rmpA_2_*	+	<20%
PB15	ST15	K21	/	*iucA*, *rmpA_2_*	−	60–70%
PB16	ST11	K64	/	*iucA*, *rmpA_2_*, *peg344*	−	60–70%
PB17	ST15	K21	/	*rmpA_2_*	+	<20%

Resistance-related genetic mutations only include those in the five genes: *phoP, phoQ, pmrA, pmrB*, and *mgrB*. The combinations of virulence genes only cover the six virulence genes detected in this study, namely *iucA*, *rmpA*, *rmpA2*, *peg344*, *iroB*, and *aerobactin*. 60–70%, 70–80%, 30–40%, and 20–30% indicate the range of results from the experiment of the triplicate.

### Genotypic antimicrobial resistance

**Figure 2:**
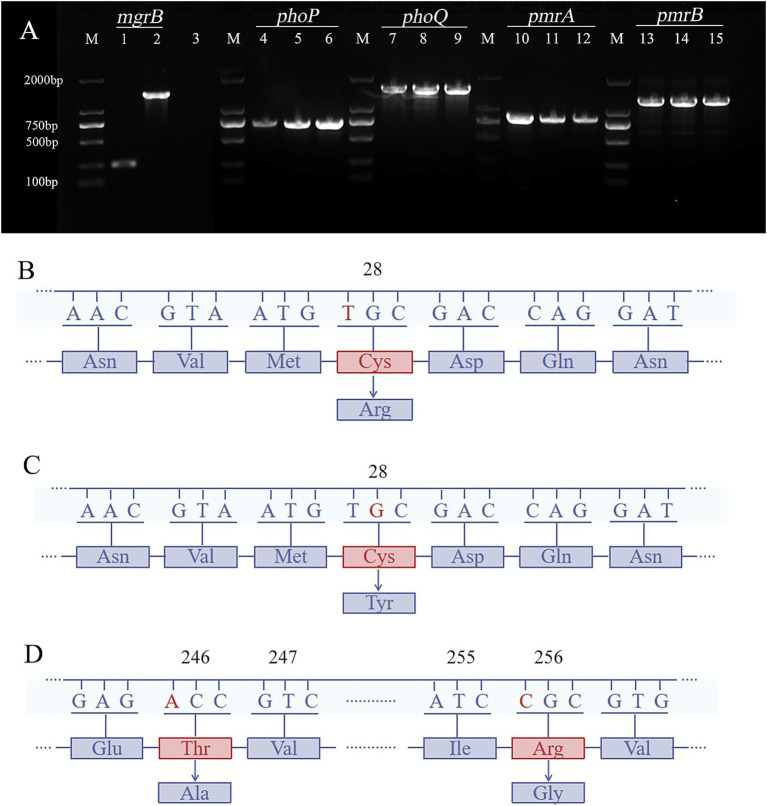
The antibiotic resistance gene profiles of 17 polymyxin-resistant *Klebsiella pneumonia* (PoRKP) strains. **(A)** Nucleic acid electrophoresis results of polymyxin-resistant genes of PoRKP. Lanes 1–2 display two distinct *mgrB* phenotypes. Lanes 4–6, 7–9, 10–12, and 13–15 represent the *phoP*, *phoQ*, *pmrA*, and *pmrB* genes in PoRKP, respectively. **(B,C)** Illustrate *mgrB* mutations (C28R or C28Y). **(D)** Shows *pmrB* mutation (T246A and R256G).

**Figure 3:**
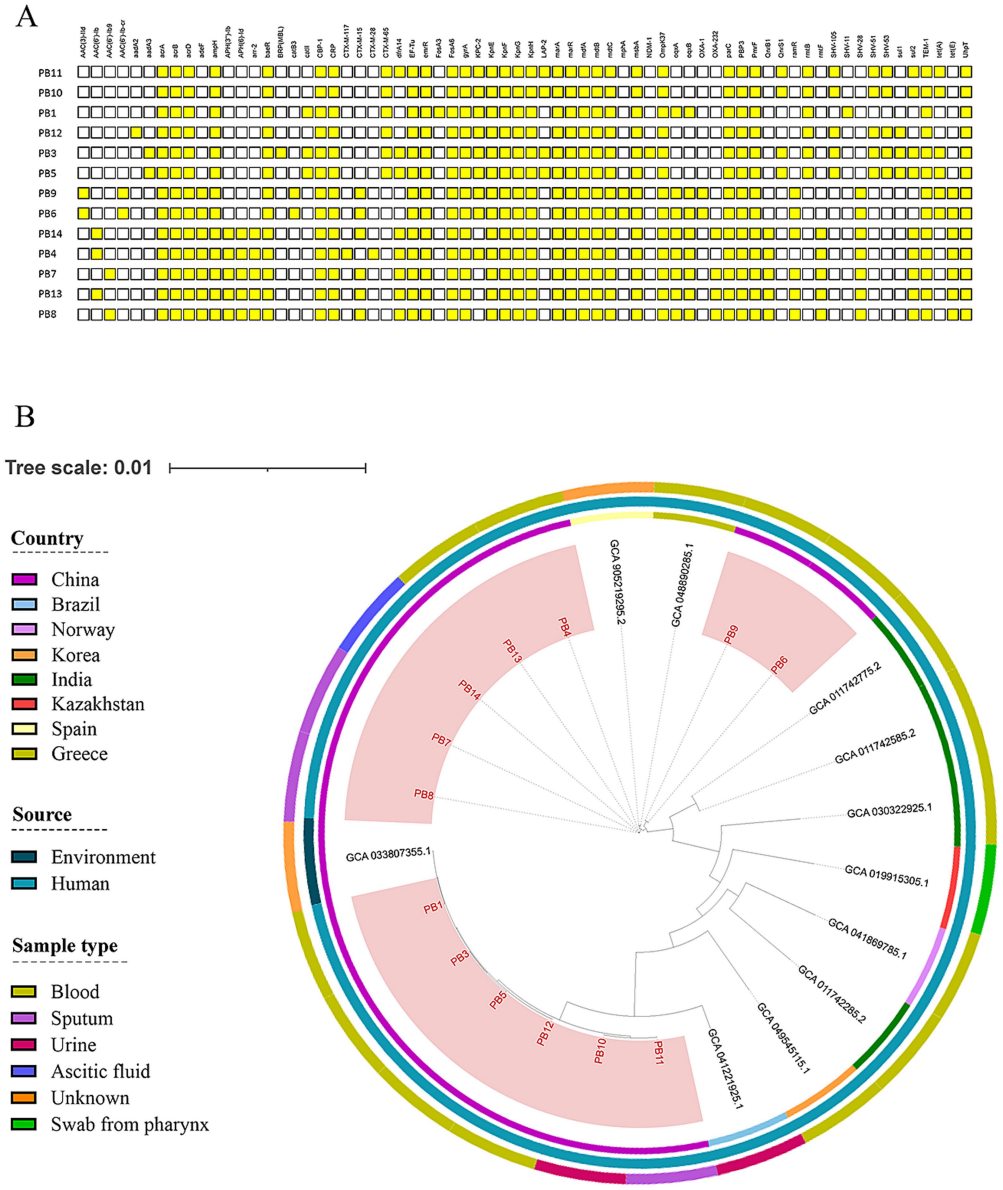
Phylogenetic analysis and other drug resistant genes. **(A)** Prediction of other drug-resistance-related genes. **(B)** A phylogenetic tree was constructed based on concatenated SNPs. The outer ring of the phylogenetic tree was categorized based on genetic distance (branch length). The inner ring displays strain names, with red indicating strains from this study, and the outer ring shows country, source species, and sample type.

PCR confirmed the presence of the resistance genes (*mgrB, phoP, phoQ, pmrA,* and *pmrB*) in all 17 clinical PoRKP isolates (Figure 2A). Furthermore, whole-genome sequencing revealed that all strains carried carbapenemase genes, among which 13 strains harbor *bla*_KPC-2_ and the remaining 4 strains (PB7, PB8, PB13, PB14) carry *bla*_OXA-232_. Notably, strain PB3 carries both *bla*_KPC-2_ and *bla*_NDM-1_ (Figure 3A).

Gel electrophoresis and sequencing results revealed no variation in *phoP* in PoRKP strains compared with the reference genome of NTUH-K2044. In three PoRKP strains, a mutation at the 28th residue of *mgrB* resulted in the substitution of the cysteine residue to either arginine or tyrosine (C28R or C28Y) (Figures 2B,C). Moreover, in six PoRKP strains, the threonine at residue 246 of PmrB was substituted by alanine, and concurrently, the arginine at residue 256 was mutated into glycine (T246A and R256G) (Figure 2D). Consequently, we hypothesized that the presence of these mutations increased the resistance of the KP isolates, consistent with previous studies (Bugli et al., 2013; Liu et al., 2021; Palmieri et al., 2020; Talat et al., 2024).

### Molecular mechanism of polymyxin resistance

Three isolates carried mobile polymyxin resistance genes, indicating that 17.6% (n = 3/17) of the PoRKP isolates were *mcr-*positive. Sequence alignment showed that three isolates, PB3 (ST11), PB7 (ST15), and PB12 (ST11), which harbored *mcr* (Supplementary Figure S1), potentially contributed to the observed variations in polymyxin MICs. All three *mcr*-positive isolates were polymyxin-resistant, with MICs of 4, 8, and 16 mg/L.

**Figure 4:**
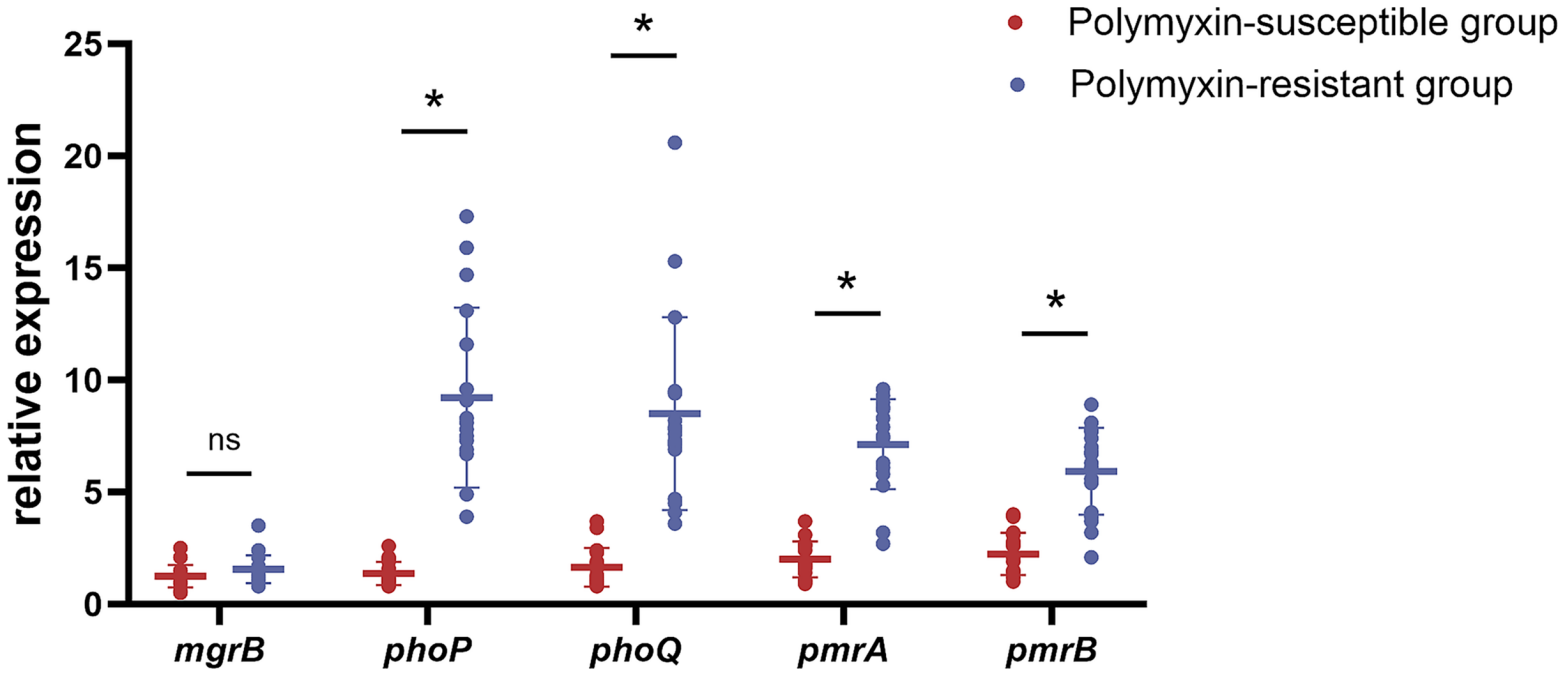
Relative expression of the *mgrB*, *phoP*, *phoQ*, *pmrA,* and *pmrB* in polymyxin-susceptible group and polymyxin-resistant group. Values were represented by means of three independent replicates. Relative expression of *phoP*, *phoQ*, *pmrA*, and *pmrB* in polymyxin-resistant group was significantly higher than polymyxin-susceptible group. There were 12 strains harbored modified-*mgrB* gene, and the rest strain were WT. *p < 0.05; ns, not significantly different.

**Figure 5:**
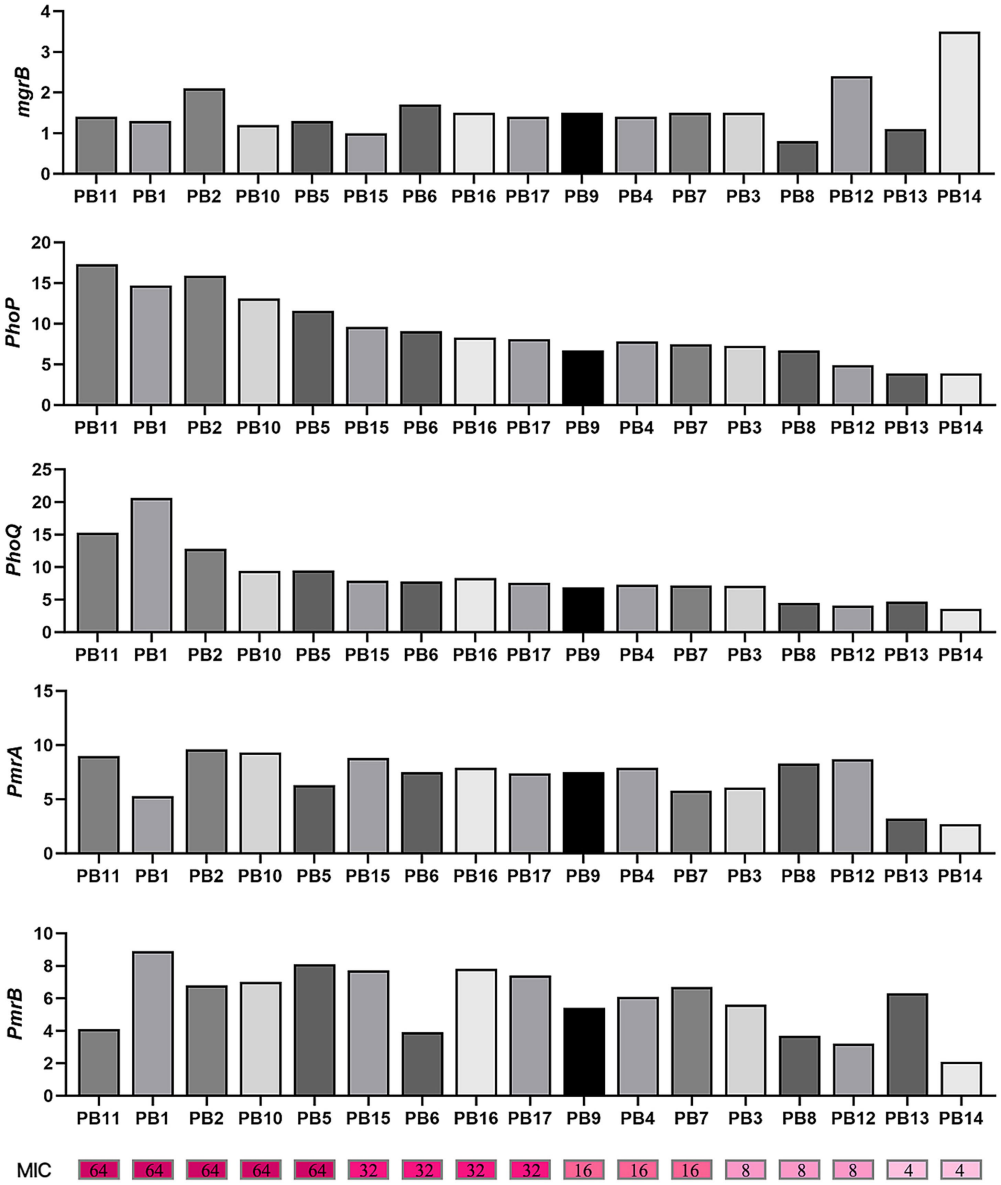
Transcriptional level of chromosomal regulators in polymyxin-resistant isolates. The top five bar plots show the transcriptional level of five chromosomal regulators, with the *x*-axis represents 17 polymyxin-resistant isolates, ordered according to their polymyxin MICs; see the color scale under the bar plot of *pmrB*.

**Figure 6:**
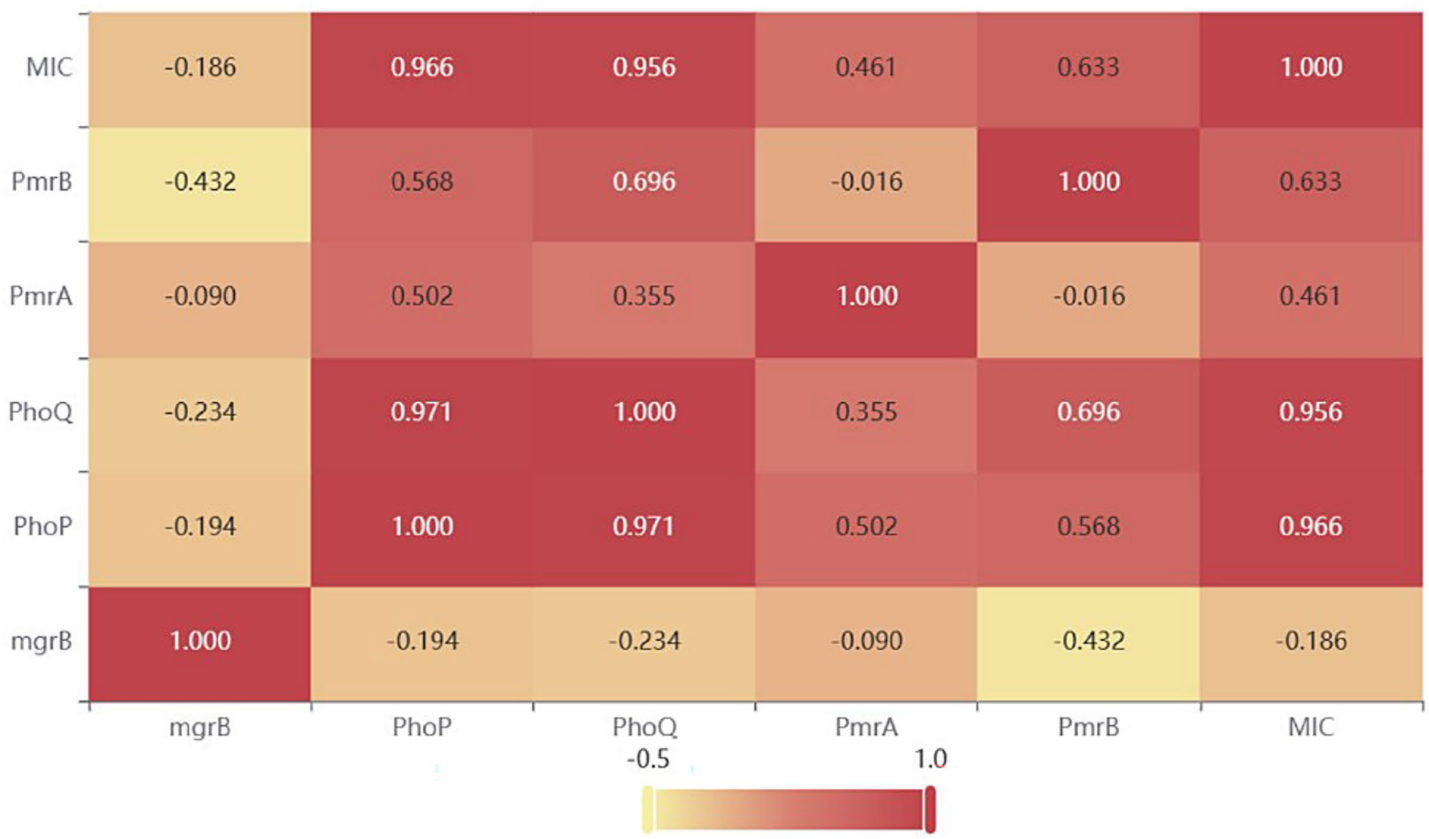
Heatmap of correlation coefficients between MIC and chromosomal regulon gene expression.

Because only three PoRKP strains harbored the plasmid-mediated polymyxin resistance gene *mcr*, we conducted further studies to understand the relationship between mutations, transcription, and polymyxin resistance based on *phoP*, *phoQ*, *mgrB*, *pmrA,* and *pmrB*. Significant differences were found between the resistant and susceptible groups in *phoP*, *phoQ*, *pmrA,* and *pmrB* (Mann–Whitney *U* test, *p* < 0.05). The expression levels of *phoP*, *phoQ*, *pmrA*, and *pmrB* in all polymyxin-resistant strains were 6.72-fold, 5.31-fold, 3.57-fold, and 2.65-fold higher, respectively, than those in polymyxin-susceptible strains (Figures 4, 5). The results were also supported by Spearman’s correlation test, as we noted a high correlation coefficient between MICs and the expression of *phoP* (0.966, *p* < 0.0001) and *phoQ* (0.956, *p* < 0.0001) (Table 4 and Figure 6).

**Table 4:** Correlation coefficient between MIC and chromosomal regulon gene expression.

Polymyxin resistance genes and MIC	mgrB	PhoP	PhoQ	PmrA	PmrB	MIC
mgrB	1 (0.000***)	−0.194 (0.456)	−0.234 (0.366)	−0.09 (0.730)	−0.432 (0.084*)	−0.186 (0.475)
PhoP	−0.194 (0.456)	1 (0.000***)	**0.971** (0.000***)	**0.502** (0.040**)	**0.568** (0.017**)	**0.966** (0.000***)
PhoQ	−0.234 (0.366)	**0.971** (0.000***)	1 (0.000***)	0.355 (0.163)	**0.696** (0.002***)	**0.956** (0.000***)
PmrA	−0.09 (0.730)	**0.502** (0.040**)	0.355 (0.163)	1 (0.000***)	−0.016 (0.952)	0.461 (0.062*)
PmrB	−0.432 (0.084*)	**0.568** (0.017**)	**0.696** (0.002***)	−0.016 (0.952)	1 (0.000***)	**0.633** (0.006***)
MIC	−0.186 (0.475)	**0.966** (0.000***)	**0.956** (0.000***)	0.461 (0.062*)	**0.633** (0.006***)	1(0.000***)

*** Correlation is significant at the 1% level. ** Correlation is significant at the 5% level. * Correlation is significant at the 10% level. The bold-faced in the table indicates a Spearman rank correlation coefficient greater than 0.5, suggesting a strong correlation.

### Prevalence of other drug-resistant genes

We analyzed other drug resistance genes in several PoRKP strains, including those related to antibiotic resistance, such as beta-lactams, aminoglycosides, and fosfomycin, as well as genes that may contribute to drug resistance, such as efflux pumps and outer membrane proteins. All strains contain more than half of these genes. (Figure 3A).

### Phylogenetic analysis of PoRKP

Phylogenetic analyses of the sequenced PoRKP isolates (BioSample accessions: SAMN50567108 (PB1), SAMN50567109 (PB3), SAMN50567110 (PB4), SAMN50567111 (PB5), SAMN50567112 (PB6), SAMN50567113 (PB7), SAMN50567114 (PB8), SAMN50567115 (PB9), SAMN50567116 (PB10), SAMN50567117 (PB11), SAMN50567118 (PB12), SAMN50567119 (PB13), SAMN50567120 (PB14)) indicated extensive genetic diversity among these strains, which were assigned to various lineages (Figure 3B).

The 17 isolates were classified as either ST11 or ST15 and were distributed across the phylogenetic tree. ST11 isolates (PB1, PB3, PB5, PB10, PB11, PB12) had a large span of SNP differences varying from 9 to 1,298, with notable examples including 32 SNPs between PB10 and PB11, and only 9 SNPs between PB3 and PB5. The ST15 strains exhibited slightly higher but still limited SNP variation (ranging from 24 to 31,775). Specifically, PB13, PB14, PB4, PB7, and PB8 showed close SNP distances (within 200), while PB6 and PB9 were slightly more distantly related to other ST15 isolates.

### Prevalence of virulence-associated genes in the isolates

**Figure 7:**
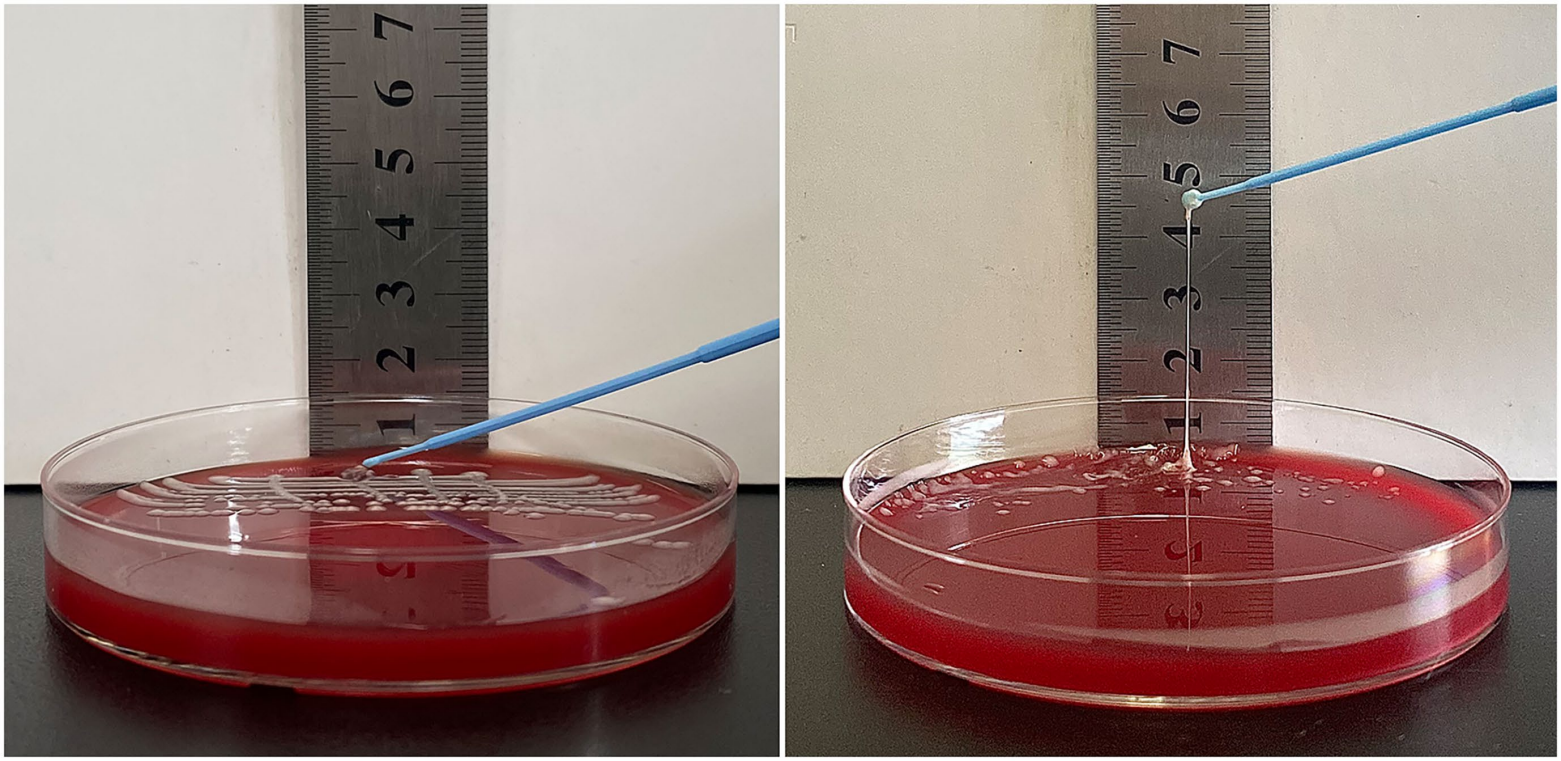
Bacterial string test. The left panel illustrates a negative string test result, while the right panel demonstrates a positive outcome.

Among the 17 PoRKP isolates, 5 (29.41%) (PB1, PB12, PB13, PB14, PB17) were positive in the string test, classifying them as polymyxin-resistant hypermucoviscous KP isolates (Figure 7).

**Figure 8:**
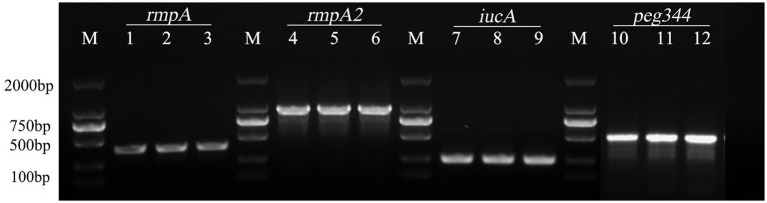
Nucleic acid electrophoresis results of virulence genes of PoRKP. Lanes 1–3, 4–6, 7–9, and 10–12 represent the *rmpA*, *rmpA2*, *iucA*, and *peg344* genes in PoRKP, respectively.

Among the 17 isolates, the virulence-associated genes included *rmpA* (35.3%, 6/17), *rmpA_2_* (82.4%, 14/17), *iucA* (76.5%, 13/17), *peg344* (47.1%, 8/17), *iroB* (0%, 0/17), and *aerobactin* (0%, 0/17) (Figure 8 and Table 3). All isolates carrying the four hypervirulence biomarkers (*rmpA, rmpA_2_, iucA* and *peg344*) belonged to ST11 (PB1, PB2, PB5, PB10, and PB12). Conversely, none of the six virulence genes were detected in isolates PB3 (ST11), PB6 (ST15), and PB9 (ST15). Based on Mantel–Haenszel *χ*₂ test, no significant correlation between virulence and MICs (*p* = 0.235) was observed, which could be attributed to the relatively limited sample size in our study.

### Virulence analysis using the *Galleria mellonella* infection model

At 72 h post-infection, seven isolates (PB1, PB7, PB8, PB12, PB13, PB14, and PB17) showed comparable virulence, resulting in 0–20% survival. Notably, only two of them have all four hypervirulence biomarkers (PB1 and PB12 isolates belonging to ST11). Based on the virulence-related information summarized earlier, we found that all string test-positive isolates (PB1, PB12, PB13, PB14, PB17) exhibited high virulence in the *G. mellonella* assay. However, whether the string test can serve as a positive marker for virulence potential requires further verification.

**Figure 9:**
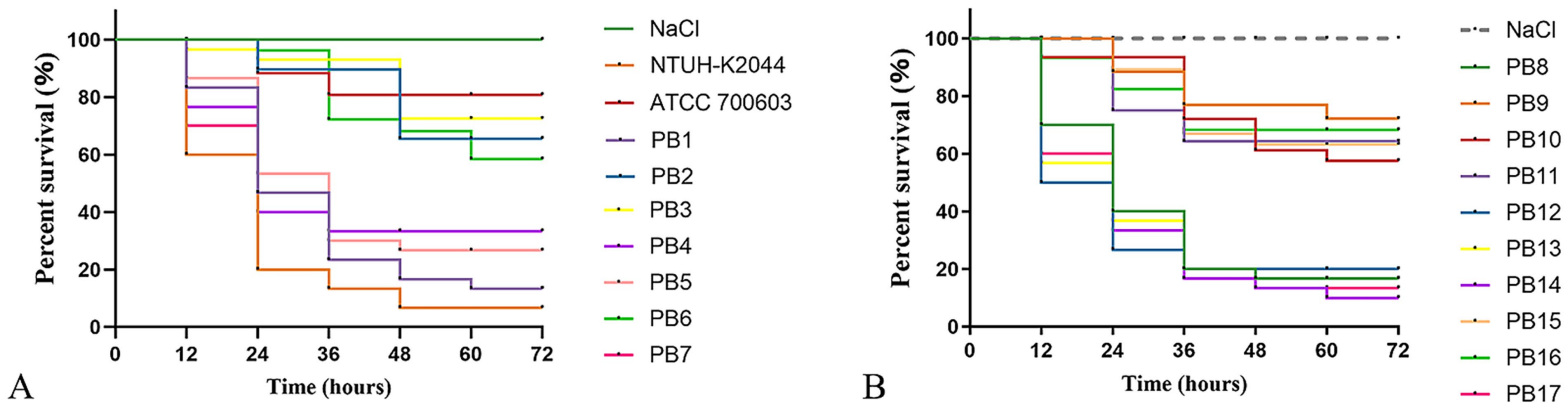
Survival curve of 3 days *Galleria mellonella l*arvae after infection with polymyxin-resistance *K. pneumonia.* **(A,B)** Present the survival data of *Galleria mellonella* following challenge with different strains. The experimental data represent the mean values from three independent replicate experiments. NaCl serves as the negative control group, while NTUH-K2044 and ATCC 700603 are the high virulence and low virulence controls, respectively.

Furthermore, the survival rates of eight isolates (PB2, PB3, PB6, PB9, PB10, PB11, PB15, and PB16) exceeded 60%. Notably, the virulence-negative control *K. pneumoniae* K6 (ATCC 700603) exhibited a survival rate of 80%, and the blank control (NaCl) showed 100% survival. The survival rates of the seven clinical isolates (PB1, PB7, PB8, PB12, PB13, PB14, PB17) were significantly lower than those of the negative control ATCC 700603, suggesting a high level of virulence (*p* < 0.001) (Figure 9).

## Discussion

Polymyxins can be used to treat infections caused by MDR-KP; however, their overuse in humans and animals contributed to the emergence of polymyxin resistance (Breijyeh et al., 2020; Mohapatra et al., 2021). In the present study, 17 polymyxin-resistant isolates were assigned to various lineages, indicating the wide genetic diversity of PoRKP. ST11 and ST15 were the dominant PoRKP STs. The percentage of XDR PoRKP strains in our study was 82.4%, indicating substantial challenges for further clinical treatment and posing a serious public health concern.

In addition, only three of the 17 polymyxin-resistant isolates carried the mobile polymyxin resistance gene, *mcr*, but exhibited different MICs for polymyxin. Compared to plasmid-mediated polymyxin resistance by *mc*r, the overexpression of the TCSs regulator genes appeared to play a more important role in resistance. Our results suggested that phoPQ regulatory factors were the primary contributor to chromosome-mediated polymyxin resistance in KP, as indicated by their strong correlation. Overexpression of TCSs, such as pmrAB and phoPQ, may lead to polymyxin resistance (Yan et al., 2021); however, it remains unclear which regulator plays the most critical role. In conclusion, chromosome-mediated resistance to polymyxins in KP is mainly caused by the upregulated expression of the phoPQ system, a key element affecting MICs.

Whole-genome analysis revealed several mutations in chromosomal regulators. Modified MgrB, mainly involving amino acid substitutions, is currently the main mechanism of polymyxin resistance in KP (Giordano et al., 2018; Shamina et al., 2020; Yang et al., 2020). PmrB (T246A and R256G), mgrB (C28Y) and mgrB (C28R) were identified in our study, which was consistent with previous research (Jian et al., 2024; Palmieri et al., 2020; Chen et al., 2023), respectively. In addition, amino acid substitutions such as T157P in PmrB (Jayol et al., 2014), D191Y in PhoP (Jayol et al., 2015), and L26P in PhoQ (Cheng et al., 2015) have been reported to increase MICs by 24-1000-folds among clinical isolates. Based on our results, we hypothesize that polymyxin resistance in KP arises from a combination of multiple mutations; however, the molecular mechanisms underlying certain amino acid substitutions remain unknown (Liu et al., 2021; Niazadeh et al., 2022; Roch et al., 2022). Further investigations are required to confirm the relationship between these mutations and polymyxin resistance.

Furthermore, we verified the virulence of the 17 PoRKP strains using the string test and the *G. mellonella* model. Seven clinical isolates exhibited relatively high virulence, with subsequent PCR analysis confirming the presence of additional virulence genes. Mutations in certain virulence genes are known to affect drug resistance (Jia et al., 2024; Li et al., 2021). For example, following bacterial biofilm formation, the biofilm impedes the contact between antibiotics and bacteria, contributing to drug resistance. It also facilitates bacterial colonization within the host and increases resistance against the attack from the host’s immune cells, thereby enhancing their virulence (Li et al., 2021). However, owing to the relatively small number of strains in our study, the correlation between drug resistance genes and virulence needs further exploration.

This study has some limitations. First, the relatively small number of strains studied may have reduced the statistical power of our findings. Second, a recent study identified a missense mutation in crrB as a contributor to polymyxin resistance (Wang et al., 2023). However, it was an accessory genome and was not detected in all polymyxin-resistant strains. Thus, the expression of crrAB system was not taken into consideration in our study. Third, the study only included samples from one hospital, which may have introduced regional bias and limited the broad generalizability of the results. Future studies with larger, more diverse cohorts and rigorous statistical adjustments are warranted to validate our findings.

In conclusion, we observed a low prevalence of *mcr* genes in PoRKP, implying that chromosome-mediated mechanisms are the primary cause of polymyxin resistance in KP. Upregulated expression of TCSs, especially phoPQ, plays a crucial role in polymyxin resistance, with *mgrB* mutations serving as the primary drivers of the overexpression of chromosomal regulators. Additionally, most PoRKPs exhibit high virulence, which could potentially be associated with the currently reported high mortality rate associated with PoRKPs. Thus, continuous surveillance of KP, especially hypervirulent PoRKP, is an urgent priority to prevent its spread.

## Data Availability

The datasets presented in this study can be found in online repositories. The names of the repository/repositories and accession number(s) can be found below: https://www.ncbi.nlm.nih.gov/genbank/, PRJNA1304718.

## Footnotes

1
http://www.pasteur.fr/recherche/genopole/PF8/mlst/Kpneumoniae.html

2
https://bigsdb.pasteur.fr/klebsiella/

3
http://blast.ncbi.nlm.nih.gov/Blast.cgi

4
https://github.com/rrwick/Unicycler

5
http://www.ebi.ac.uk/uniprot

6
http://www.genome.jp/kegg

7
http://www.ncbi.nlm.nih.gov/COG
