# Supplementary material for: Polymyxin-resistant hypervirulent Klebsiella pneumoniae clinical isolates from a tertiary hospital in China: molecular mechanisms, antimicrobial susceptibility, and virulent phenotypes
Source: Front Microbiol. 2025 Dec 17;16:1705471. doi: 10.3389/fmicb.2025.1705471 (PMC12838265; doi:10.3389/fmicb.2025.1705471)
Supplement: Supplementary file 1 [file Table_1.DOCX]

Table S1 PCR primer sequences for K-serotyping Types

| Gene name | primer sequences (5’→3’) | Amplicon Length (bp) | Annealing Temperature(℃) |
| --- | --- | --- | --- |
| *K1* | GGTGCTCTTTACATTGC | 1283 | 54 |
|  | GCAATGGCCATTTGCGTTAG |  |  |
| *K2* | GGAGCCATTTGAATTCGGTG | 1121 | 55 |
|  | TCCCTAGCACTGGCTTAAGT |  |  |
| *K5* | TGGTAGTGATGCTCGCGA | 280 | 53 |
|  | CCTGAACCCACCCCAATC |  |  |
| *K20* | CCGATTCGGTCAACTAGCTT | 1116 | 55 |
|  | GCACCTCTATGAACTTTCAG |  |  |
| *K54* | CATTAGCTCAGTGGTTGGCT | 881 | 53 |
|  | GCTTGACAAACACCATAGCAG |  |  |
| *K57* | CGACAAATCTCTCCTGACGA | 1182 | 56 |
|  | CGCGACAAACATAACACTCG |  |  |
| *K64* | TCAGTTCCGACCCTGATGCAGGTA | 268 | 54 |
| *wzi* | GCCAGAGCAACTATCATCCAAAGCCA  GTGCCGCGAGCGCTTTCTATCTTGGTATTCC  GAGAGCCACTGGTTCCAGAA(C/T)TT(C/G)ACCGC | 580 | 58 |

Table S2 PCR primer sequences for drug-resistance genes

| Gene name | primer sequences (5’→3’) | Amplicon Length (bp) |
| --- | --- | --- |
| *pmrA* | CATTTCCGCGCACTGTCTGC | 851 |
|  | CAGCTTTCAGTTGCAAACAG |  |
| *pmrB* | ACCTACGCGAAAAGATTGGC | 1274 |
|  | GATGAGGATAGCGCCCATGC |  |
| *phoP* | GAGCTTCAGACTACTATCGA | 740 |
|  | GGGAAGATATGCCGCAACAG |  |
| *phoQ* | ATACCCACAGGACGTCATCA | 1597 |
|  | CAGGTGTCTGACAGGGATTA |  |
| *mgrB* | TTAAGAAGGCCGTGCTATCC | 253 |
|  | AAGGCGTTCATTCTACCACC |  |
| *mcr-1* | CGGTCAGTCCGTTTGTTC | 309 |
| mcr-7  mcr-8  mcr-9 | CTTGGTCGGTCTGTAGGG  CAAAGAAGCGGGGTCTCCAT  CGCAGAACTGTGGGTGATCT  GGCATCTTCAACAATTCTACAAAGCG  TTTCTTCTGATGCGGCACGG  CGGCAACACCTGCAATCAAA  ATCCTTCCTGCCATCCTCCT | 112  260  121 |

Table S3 PCR primer sequences for virulence genes

| Gene name | primer sequences (5’→3’) | Amplicon Length (bp) |
| --- | --- | --- |
| *rmpA* | ACGACTTTCAAGAGAAATGA | 416 |
|  | CATAGATGTCATAATCACAC |  |
| *rmpA2* | CTGTGTCCACTATTGGTGGG | 1045 |
|  | GATAGTTCACCTCCTCCTCC |  |
| *iucA* | AATCAATGGCTATTCCCGCTG | 239 |
|  | CGCTTCACTTCTTTCACTGACAGG |  |
| *iroB* | ATCTCATCATCTACCCTCCGCTC | 235 |
|  | GGTTCGCCGTCGTTTTCAA |  |
| *peg344* | CTTGAAACTATCCCTCCAGTC | 508 |
|  | CCAGCGAAAGAATAACCCC |  |
| *aerobaction* | GCATAGGCGGATACGAACAT | 556 |
|  | CACAGGGCAATTGCTTACC |  |


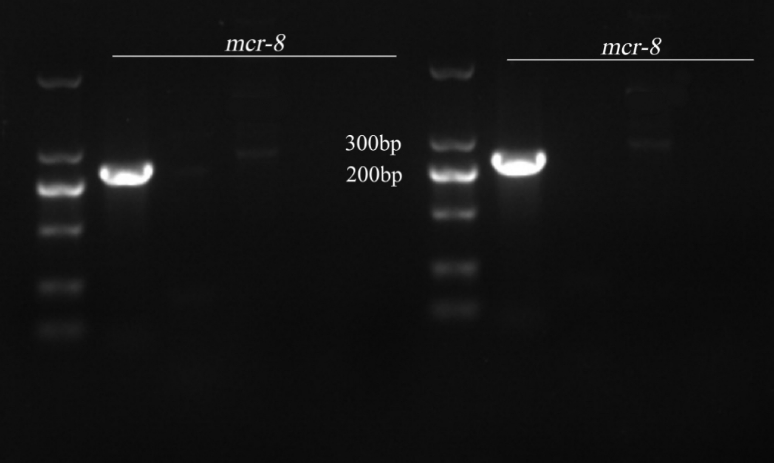


Figure S1 PCR bands of *mcr-8*
